# Fragments of *Hdh*Q150 Mutant Huntingtin Form a Soluble Oligomer Pool That Declines with Aggregate Deposition upon Aging

**DOI:** 10.1371/journal.pone.0044457

**Published:** 2012-09-12

**Authors:** David Marcellin, Dorothee Abramowski, Douglas Young, Jens Richter, Andreas Weiss, Audrey Marcel, Julia Maassen, Muriel Kauffmann, Miriam Bibel, Derya R. Shimshek, Richard L. M. Faull, Gillian P. Bates, Rainer R. Kuhn, P. Herman Van der Putten, Peter Schmid, Gregor P. Lotz

**Affiliations:** 1 Novartis Institutes for BioMedical Research, Novartis Pharma AG, Basel, Switzerland; 2 Centre for Brain Research, Auckland University, Auckland, New Zealand; 3 Department of Medical and Molecular Genetics, King’s College London, London, United Kingdom; University of Florida, United States of America

## Abstract

Cleavage of the full-length mutant huntingtin (mhtt) protein into smaller, soluble aggregation-prone mhtt fragments appears to be a key process in the neuropathophysiology of Huntington’s Disease (HD). Recent quantification studies using TR-FRET-based immunoassays showed decreasing levels of soluble mhtt correlating with an increased load of aggregated mhtt in the aging *Hdh*Q150 mouse brain. To better characterize the nature of these changes at the level of native mhtt species, we developed a detection method that combines size exclusion chromatography (SEC) and time-resolved fluorescence resonance energy transfer (TR-FRET) that allowed us to resolve and define the formation, aggregation and temporal dynamics of native soluble mhtt species and insoluble aggregates in the brain of the *Hdh*Q150 knock-in mouse. We found that mhtt fragments and not full-length mhtt form oligomers in the brains of one month-old mice long before disease phenotypes and mhtt aggregate histopathology occur. As the *Hdh*Q150 mice age, brain levels of soluble full-length mhtt protein remain similar. In contrast, the soluble oligomeric pool of mhtt fragments slightly increases during the first two months before it declines between 3 and 8 months of age. This decline inversely correlates with the formation of insoluble mhtt aggregates. We also found that the pool-size of soluble mhtt oligomers is similar in age-matched heterozygous and homozygous *Hdh*Q150 mouse brains whereas insoluble aggregate formation is greatly accelerated in the homozygous mutant brain. The capacity of the soluble mhtt oligomer pool therefore seems exhausted already in the heterozygous state and likely kept constant by changes in flux and, as a consequence, increased rate of insoluble aggregate formation. We demonstrate that our novel findings in mice translate to human HD brain but not HD patient fibroblasts.

## Introduction

A major hypothesis in Huntington`s Disease (HD) is that misfolding of mutant huntingtin (mhtt) protein leads to aggregation and causes neurodegeneration. Consistent with this idea, neurons in HD brains form intracellular inclusion bodies (IBs) composed mainly of aggregated mhtt protein. The mhtt protein present in IBs cross-reacts only with antibodies to N- but not C-terminal epitopes suggesting that mainly N-terminal mhtt fragments form IBs [1]. This is also supported by findings showing that mhtt N-terminal fragments, as compared to full-length mhtt, aggregate much more readily *in vitro* [2] and when expressed inside living cells [3]. Furthermore, intracellular proteolytic cleavage of the full-length mhtt protein [4], [5] gives rise to shorter aggregation-prone mhtt fragments while the expression of mhtt resistent to cleavage by caspase 6 is sufficient to preserve the detrimental effects of the protein on striatal volume and behavior in a YAC mouse HD model [6], [7]. Altogether, these findings strengthen the hypothesis that cleavage of full-length mhtt results in a pool of soluble toxic protein fragments that aggregate and are implicated in the pathophysiology of HD.

Quantification studies using time-resolved fluorescence resonance energy transfer (TR-FRET)-based immunoassays have revealed decreasing levels of soluble mhtt correlating with an increased load of aggregated mhtt in the aging HD mouse brain [8]. To better understand the nature of these changes at the level of native mhtt species, we developed a detection method that combines size exclusion chromatography (SEC) and TR-FRET immunoassay. Full-length and mhtt fragment complexes are separated according to size and shape and the fractions collected are further characterized using a TR-FRET based immunoassay that specifically detects soluble mhtt [8]. This TR-FRET assay uses two different fluorophore labeled-monoclonal antibodies, a terbium-labeled 2B7 antibody (2B7-Tb) that recognizes an epitope in the first N-terminal 17 aa of mhtt and a D2-labeled MW1 antibody (MW1-D2) that binds specifically to the polyQ region of mhtt [8]. A second TR-FRET assay (MW8-Tb/MW8-D2) was used to detect specifically insoluble aggregates [8]. In our previous study we used both TR-FRET assays to establish an inverse correlation between soluble and aggregated mhtt in *HdhQ*150 brains [8]. However, we were not able to discriminate mhtt species in larger protein complexes containing either fragments and/or full-length mhtt. In this study the combination of SEC and TR-FRET (SEC-FRET) allowed us to unmask previously unrecognized age- and gene-dosage dependent changes in abundance and heterogeneity of native soluble mhtt complexes.

Here, we demonstrate that the *Hdh*Q150 mouse brain harbors a pool of mhtt fragment-oligomers whereas full-length htt remains monomeric. The mhtt fragment-oligomer pool is already present in the brain of one month-old *Hdh*Q150 mice, long before disease phenotypes and mhtt aggregate histopathology appear. We demonstrate, that the pool-size changes of soluble mhtt fragment-oligomers correlate inversely with the accumulation of insoluble aggregates in an age-dependent manner whereas the levels of soluble full-length mhtt protein remain stable as the mice age. The formation of insoluble aggregates is most prominent in the striatum and markedly accelerated in a dose-dependent fashion in homozygous as compared to heterozygous *Hdh*Q150 mice. In contrast, the pool-size of soluble mhtt oligomers seems mhtt allele-dose independent which seems in line with the hypothesis that flux-rate of mhtt fragments through this pool but not its size might be rate-limiting in the formation of insoluble IBs [9]. The importance of our findings with respect to the disease in humans is further emphasized by showing the existence of a mhtt fragment-oligomer pool in a human HD brain. Therefore, applying SEC-FRET analysis may become a novel and important cornerstone methodology to identify disease-modifying targets in HD that directly affects mhtt fragmentation or aggregation.

## Materials and Methods

### Ethic Statement

All experiments were carried out in accordance with authorization guidelines of the Swiss Federal and Cantonal veterinary offices for the care and use of laboratory animals. Studies described in this report were approved by the Swiss Cantonal veterinary offices (Basel-City) and preformed according to Novartis animal license number BS-1858.

### Animals

Heterozygous *Hdh*Q150 knock-in mice [10] were obtained from the laboratory of G. P. Bates. *The Hdh*Q150 mice were maintained on a C57BL/6J background. The offspring were genotyped by polymerase chain reaction (PCR) using DNA obtained from ear punches. The animals were housed in a temperature-controlled room that was maintained on a 12 h light/dark cycle. Food and water were available *ad libitum.* Animals were sacrificed by decapitation in deep isoflurane narcosis. Tissues were then collected immediately and were snap-frozen on a metal plate placed on dry ice.

### Cell Lines

Mouse embryonic stem (ES) cells expressing human exon1 or full-length mhtt (Q145) from the ROSA26 locus were cultivated in 3i–medium [11], [12]. Neurons were derived from ES cells as described [13]. These cells differentiated to glutamatergic pyramidal neurons and were kept in culture for 21 days.

### SV 40 Immortalized Human HD Fibroblasts and Human HD Brain

Fibroblasts were received from Coriell Institute for medical research, New Jersey, USA [Code GM04729 and GM04723]. Fibroblast cells expressing wildtype Q17 or mutant Q68 were cultured in MEME - medium (Sigma, cat# M-5650) +15% FCS (Gibco, cat# 10270) +5 ml Glutamax (Gibco, cat# 35050) +5 ml PenStrep (Gibco, cat# 15140) and were grown on 15 cm dishes (1.5×10^6^) until confluence. Human brain tissue was obtained from the Neurological Foundation of New Zealand Human Brain Bank.

### Western Blotting Analysis

Cells were harvested and lysed in ice-cooled lysis buffer (phosphate buffered saline, Invitrogen #14190; 1% Triton X-100), containing anti-proteases (Roche, cOmplete, Mini Protease Inhibitor Cocktail Tablets, 04693124001) and anti-phosphatases (Roche, PhosStop Phosphatase Inhibitor Tablets, 04906845001). Lysates were kept on ice for 15 min before to be completely homogenized by sonication for 5 seconds. A BCA assay (Thermo Scientific, 23227) was used for protein quantification. Samples were diluted in NuPAGE loading buffer (Invitrogen, NP0007; NP0009) and heated 10 min at 95°C. The samples were loaded onto 4–12% NuPAGE Bis-Tris gels or 3–8% Tris-acetate gels (Invitrogen, NP0335, EA03752). Proteins were transfered to PVDF membranes for 10 minutes with the iBlot Dry Blotting System (Invitrogen, IB-4010-01) according to manufacturer instructions. The membrane was then incubated for one hour in phosphate buffered saline, 0.1% Tween 20, 5% (w/v) dried milk. Incubation with primary antibodies was done over-night (O/N) at 4°C; before secondary horseradish peroxidase conjugated goat anti-mouse (GE Healthcare, RPN4201) were applied for one hour. Protein bands were detected with chemiluminescent substrate (ECL, GE Healthcare).

### Antibodies

MW1 and MW8 antibodies were developed by Paul Patterson [14] and obtained from the Developmental Studies Hybridoma Bank, developed under the auspices of the NICHD and maintained by The University of Iowa, Department of Biological Sciences, Iowa City, IA 52242. Generation and characterization of 2B7 was described previously [15].

### Immunostaining

10 µm frozen sagittal sections were prepared from snap frozen brain hemispheres, placed onto SuperFrost plus glass slides (Menzel) and air dried for 10 minutes. Sections were then fixed for 30 minutes at room temperature with 4% paraformaldehyd/PBS pH 7.4, rinsed 3 times for 5 minutes in PBS, incubated for 1 hour with PBS containing 2% goat serum, reacted over night at 4°C with 3 µg/ml Alexa 488-labeled MW8 diluted in PBS containing 2% goat serum, washed 3 times in PBS and mounted with Prolong Gold containing DAPI nucleic acid counterstain (Invitrogen). To increase staining intensity, some slides were reacted over night with 3 µg/ml unlabeld MW8, rinsed in PBS and incubated for 1 hour at room temperature with Alexa 594-labeled secondary antibody (Invitrogen) before beeing mounted. Quantitative evaluation of aggregate size was performed with CellF (Olympus Soft Imaging Solutions) software.

### Size Exclusion Chromatography (SEC): Analysis of Soluble mhtt by SEC-FRET

All samples were prepared in 500 µl of complete ice-cooled lysis buffer [phosphate buffered saline, (Invitrogen #14190), 1% Triton X-100, anti-proteases (Roche, cOmplete, Mini Protease Inhibitor Cocktail Tablets, 04693124001) and anti-phosphatases (Roche, PhosStop Phosphatase Inhibitor Tablets, 04906845001)]. *Hdh*Q150 brain tissues (forebrain, striata pool) were homogenized with ceramic beads (Precellys, Bertin Technologies) according to the manufacturer instructions and finally sonicated for 10 seconds. Cells (ES lines and human fibroblasts) were resuspended in complete ice-cooled lysis buffer and sonicated for 10 seconds. Human post-mortem brain cortices were provided already homogenized in complete lysis buffer. Extracts were then clarified by ultra-centrifugation for 30 min at 100,000 g. Supernatants were filtered through a 0.45 µm membrane (Millipore, Millex PTFE) and protein concentrations were determined by BCA. Proteins were injected and fractionated by SEC on a Superdex 200 10/300 column. The total protein loaded on the column in a sample volume of 500 µl were respectively, 300 µg for the ES cell lines, 3.0 mg for the *Hdh*Q150 forebrain and striata pool, 4.0 mg for the human fibroblasts and 1.0 mg for human post-mortem brain cortices. The separation was performed at 4°C with a flow rate of 0.5 ml/min in PBS 0.05% Triton X-100. The elution was done with one column volume and fractions (250 µl per fraction) were collected in 96-well plate format and 10µl from each were applied for TR-FRET measurement with indicated antibody combinations. Protein standards were used to estimate size of htt peaks (**Fig. S1A**).

### Analysis of Insoluble mhtt Aggregates by TR-FRET

Pellet from *HdhQ150* brain homogenate obtained by ultra-centrifugation at 100000 g (density separation) were washed once with PBS +1%Triton X-100 and resuspended in the same buffer and sonicated. 10 µl of the pellet suspension were assayed for TR-FRET measurement with MW8-Tb/MW-8-D2 as previous described (8). Protein concentration was determined for normalization.

### Time-resolved Förster Resonance Energy Transfer (TR-FRET)

Assays were performed as described previously [16], [17], [18]. Briefly, 10 µl of sample (lysate or SEC fractions) was transferred to a low-volume plate (Greiner Bio-one, #784080) and 1 µl of antibody mix added. Soluble mhtt was measured with 2B7-Tb/MW1-D2. Aggregated mhtt was measured with MW8-Tb/MW8-D2. Fluorophore labeling of antibodies was performed by CisBio Bioassays (Parc Marcel Boiteux, France). TR-FRET measurements were done after over night incubation at 4°C using an EnVision Reader (Perkin Elmer, excitation 320 nm, time delay 100 msec; integration time 400 msec). Values are expressed as the ratio between the emission at 665 nm and 620 nm. Each ratio was then expressed in Delta F: the sample ratio was first reduced with the background signal ratio (antibodies in lysis buffer), then divided by the background signal ratio and finally multiplied by 100. Delta F values were then normalized to protein concentration in the case of brain tissues**.**

## Results

### Detection of Soluble mhtt Protein Fragments in Embryonic Stem Cell-derived Neurons Expressing Full-length mhtt

**Figure 1:**
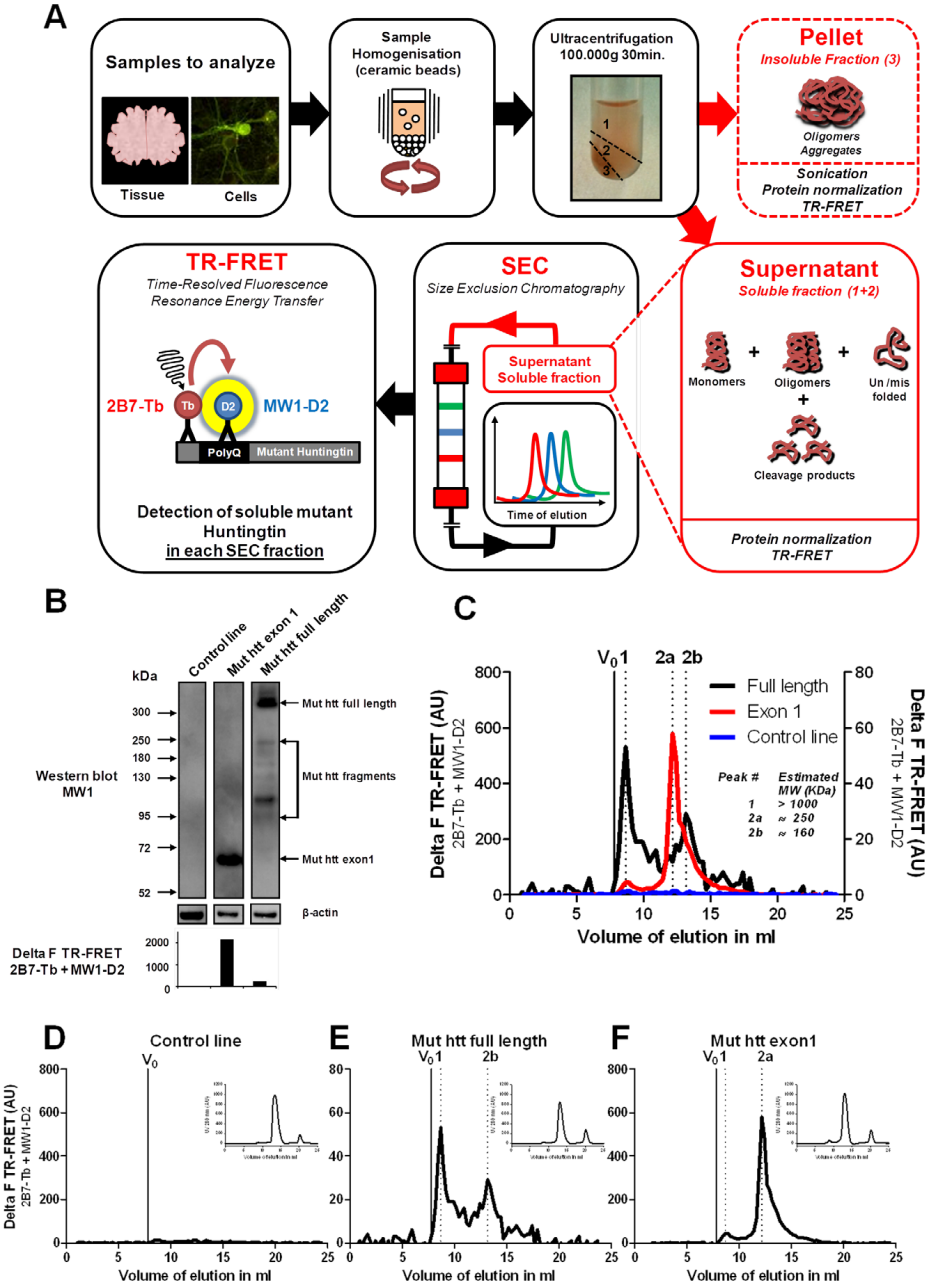
The SEC-FRET technology is a useful tool to detect multiple mhtt species in cell lysate. (A) Schematic overview of the SEC-FRET methodology. (B) Detection of multiple mhtt fragments in lysate of neurons derived from mouse ES cells by Western blot analysis with MW1 antibodies (C) Overlap of all TR-FRET-based detection profiles after SEC (full-length in black, exon1 in red and wt in blue) Estimated MW of TR-FRET peaks (1, 2a and 2b) based on standard marker in Fig. S1. AU: arbitary units. (D, E, F), Individual TR-FRET based detection profiles after SEC with the UV absorbance, as an indicator of protein concentration, in the upper right corner for stem cell derived neurons at 21 days *in-vitro* (D) control line, (E) mhtt full length (Q145) and (F) mhtt Exon1 (Q145).

To determine the existence, the size and the relative abundance of different soluble mhtt protein fragments in cells expressing a full-length mhtt protein, we first analyzed cell lysates of mouse ES cell-derived neurons that over-express a human full-length mhtt protein with a polyQ length of 145 (145Q). The mhtt gene is inserted into the ROSA26 locus [11], [12]. Western Blot analysis of these cell lysates identified several mhtt protein fragments ranging in size between 90 and 250 kDa and indicating that cleavage of the full-length mhtt protein occurs in these neurons (**Fig. 1B**). To characterize in more depth the native biochemical and molecular features of these mhtt protein fragments in solution, we separated them from mhtt full-length proteins by size-exclusion-chromatography followed by TR-FRET-based analysis of the different fractions (**Fig. 1A**). The TR-FRET- immunoassay we used, specifically detects soluble mhtt and not wildtype htt or insoluble mhtt [8] (**Fig. 1A**). After injection of 300 µg total soluble protein into the size exclusion column, full-length mhtt protein is detected in fractions of molecular weights around 1000 kDa (peak1 in **Fig. 1C, E**). Interestingly, another fraction containing soluble mhtt appeared around 160 kDa (peak 2b in **Fig. 1C,E**) suggesting it might contain oligomerized mhtt fragments. Analysis of wildtype ES cell extracts which contain only endogenous mouse wildtype htt protein revealed no FRET signals above background confirming the specificity of the 2B7-Tb/MW1-D2 TR-FRET assay detecting only mhtt but not wild-type htt species (**Fig.1** **C and D**) [**8**].

Next, we ran SEC-FRET assays also on extracts of neurons derived from mouse ES cells that over-express mhtt human exon1 (Q145) located in the ROSA26 locus. A key question we wanted to address was whether exon1-derived mhtt protein species differ in their biochemical nature and size from mhtt fragments generated by cleavage of full-length mhtt. As shown in **Fig. 1C and 1F**, the peak profile of the 250 kDa fractions of the exon1 cell extract only partially overlaps with that of the 160 kDa fractions in the ES-derived neurons expressing full-length mhtt. Western blot analysis revealed that the exon1-derived mhtt fragments differ from those generated in the cells expressing full-length mhtt (**Fig. 1B**). We also noted that the 2B7-Tb/MW1-D2 TR-FRET assay detects fragments with much greater sensitivity (about 10 fold) than mhtt full-length (**Fig. 1B and C**). Note, total mhtt protein expression levels in the two ES-derived neuronal cell types were similar (**Fig. 1B**). Altogether, the findings in these two ES-derived neuronal cell populations suggest that mhtt fragments and the soluble complexes they form, differ when derived from full-length cleavage as compared to exon-1 mhtt. Interestingly, the formation of mhtt-containing aggregates is only observed in the exon-1 mouse ES cell model but not in its counterpart expressing full-length mhtt (data not shown). Whether this relates to different intracellular fragment concentrations achieved in the two cellular systems and/or to their biochemical nature requires further clarification. Taken together, the results in these two cellular models demonstrate that SEC-FRET is a valid and useful methodology to further characterize the size, biochemical nature and relative abundance of soluble complexes of mhtt fragments in different HD-relevant biological systems.

### An Invariant Soluble Pool of mhtt Oligomers in the *Hdh*Q150 Mouse Brain is Rate-limiting to IB Formation

**Figure 2:**
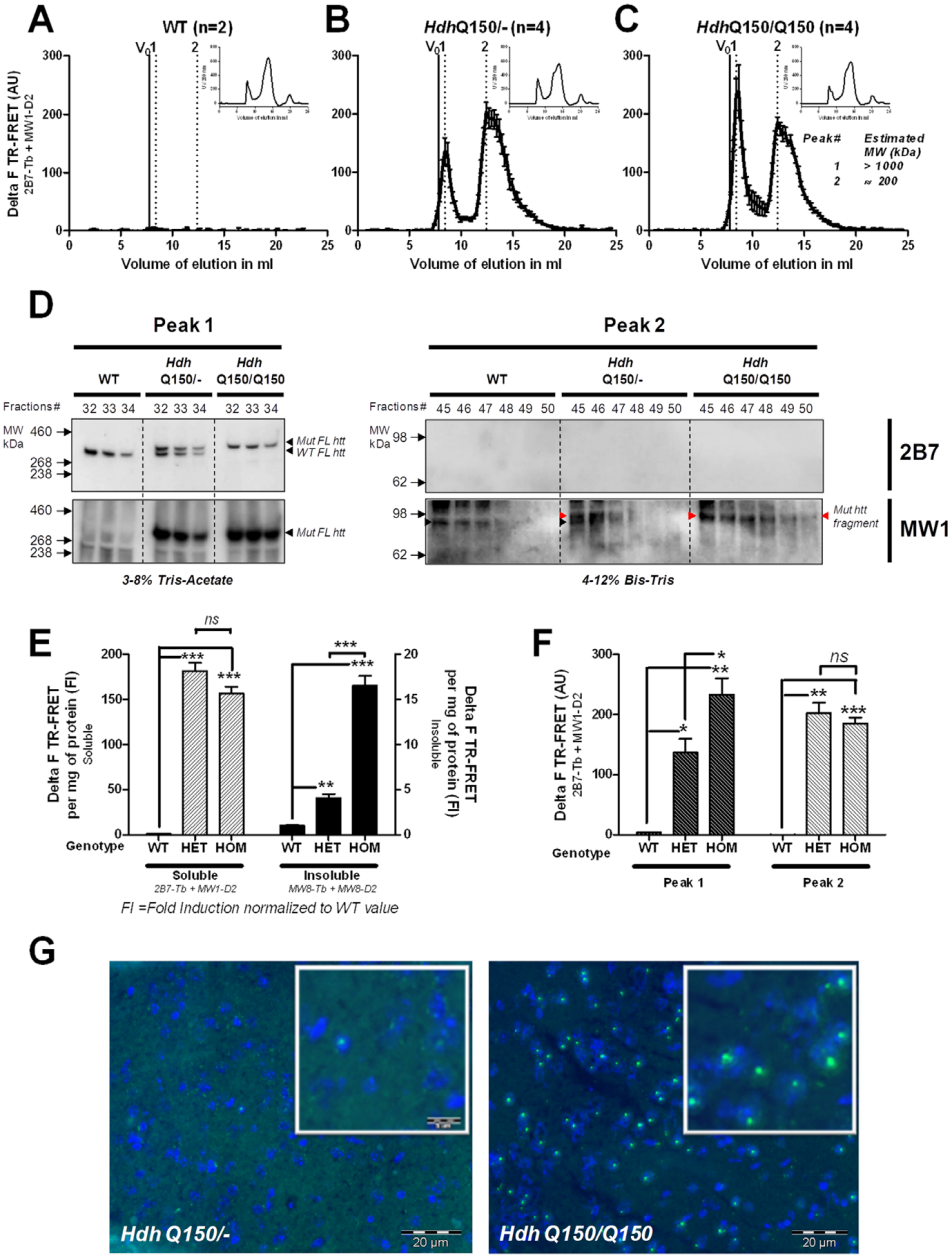
Analysis of 8 months *Hdh*Q150 forebrain identified invariant, soluble oligomeric pool of mhtt fragments. (A), (B), (C), Individual TR-FRET based detection profiles after SEC with the UV absorbance in the upper right corner for A: Wild type, B: Heterozygote *Hdh*Q150, C: Homozygote *Hdh*Q150 forebrain samples at 8 months. Molecular weight of TR-FRET peaks are indicated in (C). (D) Western Blot analysis to detect htt full-length in the fractions 32, 33, 34 for peak1 and mhtt fragments at kDa (red arrow) in fractions 45, 46, 47, 48, 49, 50 for peak 2 using either 2B7 or MW1 antibodies. Note, black arrow in wt (peak2) for MW1 detection is non-specific background and below the specific mhtt fragment band in *Hdh Q150*/− and *Hdh Q150/Q150* (E) Quantification of soluble mhtt by 2B7-Tb/MW1-D2 TR-FRET detection in supernatant fraction and of insoluble mhtt by MW8-Tb and MW8-D2 detection in resuspended and sonicated pellet fraction taken from wild type, *Hdh Q150*/− and *Hdh Q150/Q150* forebrain tissue. Delta F values (see experimental procedure) are mean of 4 independent measurements ± SEM. Delta F values are adjusted to protein concentration and then normalized to wild-type. The t-test was used to assess significance (ns P>0.05; * P<0.05; ** P<0.01, *** P<0.001) (F) Quantification of soluble mhtt in peak1 and peak 2 by 2B7-Tb/MW1-D2 TR-FRET detection for wild type, *Hdh Q150*/− and *Hdh Q150/Q150*. Delta F values (mean of 4 independent measurements ± SEM) are expressed as the maximal peak value. The t-test was used to assess significance (G) Immunostaining of aggregated mhtt in the striatum of 4 months old heterozygote (left) and 4 months old homozygote *Hdh*Q150 mice (right). Frozen sections were stained with Alexa 488-labeled MW8 and counterstained with DAPI to visualize cell nuclei (blue fluorescence).

SEC-FRET was used to characterize mhtt protein products in forebrain extracts of 8 month-old *Hdh*Q150 knock-in mice expressing endogenous full-length mhtt protein that is known to generate multiple proteolytic cleavage products [19]. In heterozygous mice, SEC-FRET analysis using the 2B7-Tb/MW1-D2 antibody pair revealed a full-length mhtt species in the 1000 kDa fraction (peak1) and one major lower molecular weight peak at around 200 kDa (peak2) (**Fig. 2B**), indicating that in these mice mhtt oligomeric fragment assemblies exist similar to those we detected in ES-derived neurons that over-express full-length mhtt. Western Blot analysis confirmed the presence of full-length mhtt in the 1000 kDa peak 1 fractions and mhtt fragments in the 200 kDa (peak 2) fractions (**Fig. 2D**). Note, Western blot analysis with the 2B7 antibody did not enable the detection of mhtt fragments in the peak 2 SEC-fractions because the concentration of 2B7 cross-reacting fragments is too low. In contrast, Western blot analysis of the total supernatant fraction (100.000 g) before it was loaded onto the SEC column did show mhtt fragments of around 80 kDa (**Fig. S1B**). Note, these were slightly different in size from the mhtt fragments detected in peak 2 with MW1 demonstrating that heteromeric mhtt complexes with monomers of different lengths exist in the peak 2 fractions (**Fig. S1B**). Wild-type mice did not show any signals (**Fig. 2A**).

Unlike longer CAG repeats, homozygosity for the HD mutation in patients appears not to lower significantly the age at onset of symptoms [20]. Rather, doubling the dose of the mhtt allele appears to influence result mainly in a more severe clinical course with the note of caution that the number of homozygous patients analyzed remains low [20]. In mouse models, increasing the expression of transgenes has been shown to accelerate both the onset and the progression of phenotypes [6], [21], [22], [23]. Thus, we compared mhtt (fragments *vs* full-length and soluble oligomers *vs* insoluble aggregates) in age-matched heterozygous and homozygous *Hdh*Q150 mouse brains using immunohistochemistry, TR-FRET, SEC-FRET and Western Blot analysis. The formation of neuronal MW8-positive IBs in the striatum is markedly exacerbated in homozygous as compared to heterozygous *Hdh*Q150 mice as shown in brain sections of 4 month-old mice (**Fig. 2G**). Analysis of forebrain extract using TR-FRET to detect soluble mhtt species: (2B7-Tb/MW1-D2 forebrain extract supernatant, 100,000 g) and insoluble mhtt aggregates (MW8-Tb/MW8-D2: sonicated pellet), revealed no significant differences in the levels of soluble mhtt in hetero- versus homozygous mice. However, homozygous mice showed a highly significant increase in insoluble aggregates (**Fig. 2E**) in line with our immunohistochemical observations (**Fig. 2G**). Most importantly, these results demonstrate that increased gene-dosage of mhtt in homozygous mice greatly accelerates insoluble aggregate formation *in vivo* (**Fig. 2E, G**).

SEC-FRET analysis comparing hetero- and homozygous mice revealed that the relative abundance and heterogeneity of soluble mhtt fragments did not change but, as expected, the amount of full-length mhtt increased in the homozygous brain (**Fig. 2B, C, F**). Western Blot analysis confirmed that mhtt full length protein elutes at 1000 kda (peak1) and mhtt fragments at around 200 kda (peak2) (**Fig. 2D**). Remarkably, the profile of oligomeric mhtt fragments in the 200 kDa fractions of hetero- and homozygous mice did not significantly differ despite the two-fold higher levels of full-length mhtt expressed in the homozygotes, the earlier onset and more numerous formation of IBs (**Fig. 2B, C, F, G, E**). These results suggest that the pool of mhtt fragment-oligomers is remarkably constant with respect to size and profile and largely mhtt gene-dosage independent. On the other hand, gene-dosage is rate-limiting to IB formation in the mouse *in vivo* as it was in cultured cells [**9**]. Altogether, these results seem to fit best with a mhtt cleavage pathway model in which the maximal capacity of an oligomeric mhtt fragment pool is reached and kept constant by dynamics in flux-rate.

### Age-dependent Inverse Correlation between Soluble and Aggregated mhtt Fragment-pools

**Figure 3:**
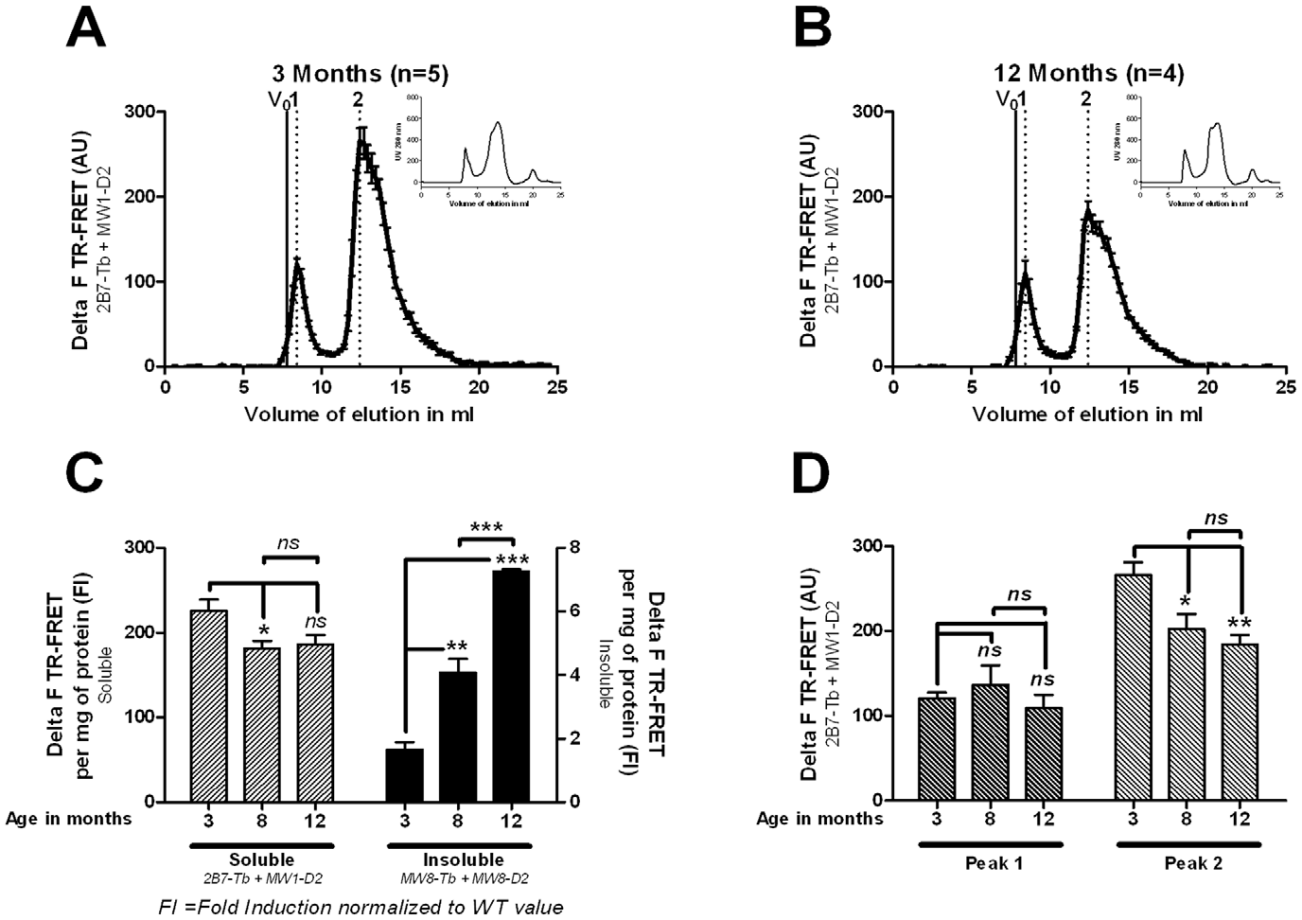
Inverse correlation of soluble and aggregated mhtt fragments analyzed by *Hdh*Q150/− forebrain tissue. SEC-FRET profiles with the UV absorbance in the upper right corner for (A) 3 months and (B) 12 months heterozygote *Hdh Q150/−* forebrain tissue. (C) Quantification of soluble mhtt by 2B7-Tb and MW1-D2 TR-FRET detection in supernatant fraction and of insoluble mhtt by MW8-Tb and MW8-D2 detection in resuspended pellet fraction of *Hdh Q150*/− mice at 3, 8 and 12 months. Delta F values (see experimental procedure) are mean of 4 independent measurements ± SEM. Delta F values are adjusted to protein concentration and then normalized to wild-type. The t-test was used to assess significance (D) Quantification of SEC fractions of peak 1 and of peak 2 by 2B7-Tb and MW1-D2 TR-FRET taken from *Hdh Q150*/− mice at 3, 8 and 12 months. Delta F values (mean of 4 independent measurements ± SEM) are expressed as the maximal peak value; The t-test was used to assess significance.

To determine age-dependent changes in SEC-FRET profiles, we analyzed forebrain extracts of heterozygous *Hdh*Q150 mice at the age of 3, 8 and 12 months (**Fig. 3A-D**). Identical protein concentrations (confirmed by UV absorption) were loaded. Accordingly, we could show that the pool size of oligomeric mhtt fragments in the 200 kDa peak fractions decreased over time as the mice aged (**Fig. 3** **A, B, D**). In contrast, the levels of full-length mhtt in the 1000 kDa fractions remained constant (**Fig. 2B** **;** **Fig. 3A, B, D**) whereas the amounts of insoluble aggregates increased dramatically with age (MW8-Tb/MW8-D2 TR-FRET; **Fig. 3C**).

Using supernatant of forebrain extract (100000 g), soluble mhtt levels were determined by 2B7-Tb/MW1-D2 TR-FRET assay (**Fig. 3C**). The TR-FRET analysis revealed a significant decrease of soluble mhtt occurring between 3 and 8 months of age but no further significant decline between 8 and 12 months (**Fig. 3C**). Strikingly, peak-specific SEC-FRET analysis of the 1000 kDa and 200 kDa fractions confirmed a significant age-dependent decrease between 3 and 8 months in the amounts of soluble mhtt fragments (200 kDa fraction/peak 2) but not in the amount of full-length mhtt (1000 kDa fraction/peak 1) (**Fig. 3D**). Altogether, these findings indicate that the pool of mhtt oligomerized soluble fragments but not the pool of full-length mhtt protein correlates inversely with the formation of insoluble mhtt protein aggregates in *Hdh*150Q mouse forebrain. The inverse correlation is most pronounced in the 3–8 month age interval after which the size of the 200 kDa soluble pool remained at a steady state.

### Inverse Correlation of Soluble and Aggregated mhtt Fragments is Most Prominent in the Striatum

**Figure 4:**
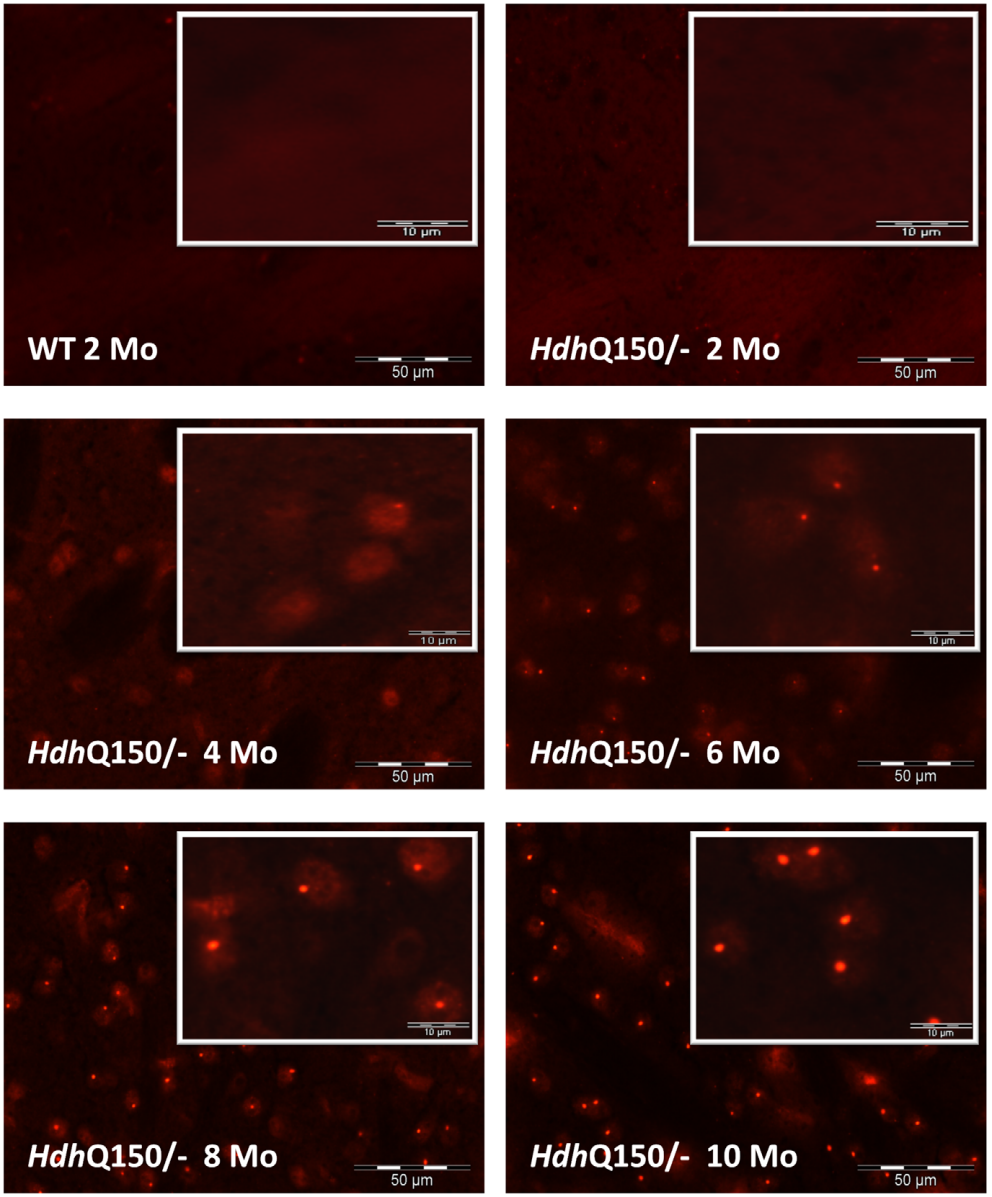
Immunostaining of aggregated mhtt in the striatum of *Hdh*Q150/− mice. Frozen sections were stained with unlabeled MW8 and Alexa 594-labeled secondary antibody. In wt control and at 2 months of age only non-specific background fluorescence is visible. At 4 months of age diffuse granular staining is detectable, predominantly in the nucleus. At 6 months of age the diffuse nuclear staining is less pronounced and small nuclear inclusions are visible. At later stages the nuclear inclusions are significantly larger and the granular staining is strongly diminished at 10 months of age. Note, the large elongated and irregular shaped fluorescent structures visible in images taken from *Hdh*Q150**/−** mice at 8 and 10 months of age are blood vessels (non-specific staining of mouse IgG by the secondary antibody).

**Figure 5:**
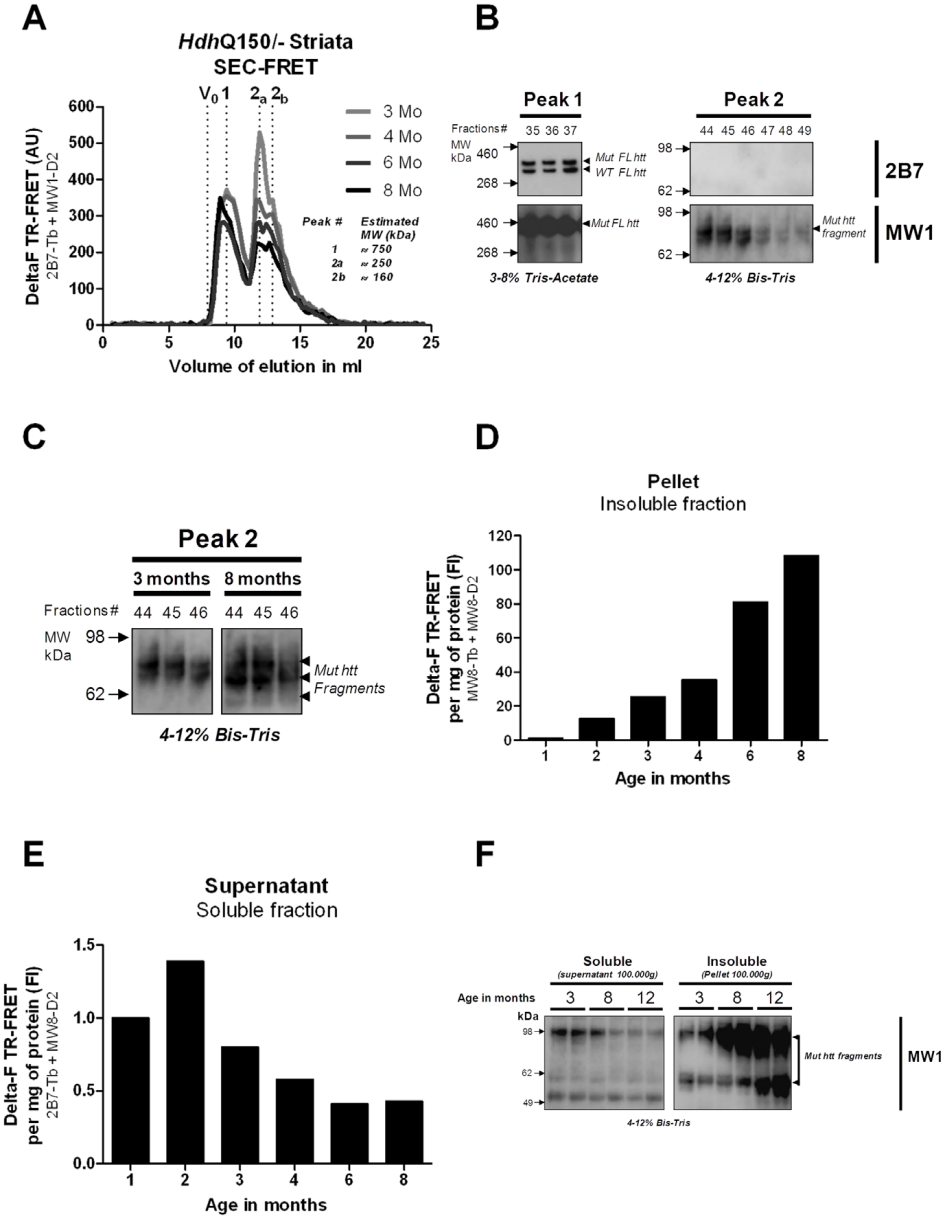
Analysis of *Hdh*Q150/− striatum. (A) Overlapped SEC-FRET profiles at 3, 4, 6 and 8 months of *Hdh*Q150/− striatum samples. (B) Detection of htt full-length protein (FL) by Western blot analysis in fractions 35–37 (peaks 1) and of mhtt fragments in fractions 44–49 (peak 2) using either 2B7 or MW1 antibodies. (C). Detection of mhtt fragments by Western Blot analysis of fractions 44, 45, 46 of *Hdh*Q150/− striatum samples at 3 and 8 months (D) Quantification of the insoluble mhtt by TR-FRET MW8-Tb and MW8-D2 detection. (E) Quantification of the soluble mhtt by TR-FRET 2B7-Tb and MW8-D2 detection. (F) Detection of mhtt fragments by Western Blot analysis of soluble (supernatant, 100000 g) and insoluble (pellet) brain extracts of *Hdh*Q150/− mice at 3, 8 and 12 months.

The striatum is the most severely affected brain region in HD patients [24], [25] and also the earliest and most profoundly affected brain region in *Hdh*Q150 mice [10]. Thus, we analyzed striata of heterozygous *Hdh*Q150 mice at the ages of 2, 4, 6, 8 and 10 months by immunohistochemistry and at 1, 2, 3, 4, 6 and 8 months using the SEC-FRET method (**Fig. 4** **and** **5**). Immunohistochemical analysis using the MW8 antibody showed that the number and size of intranuclear IBs in the striatum gradually increased with age (**Fig. 4**). Note, that after the age of 8 months, mainly aggregate size but not their number increased significantly (see 8 as compared to 10 month-old immunostaining quantification; **Fig. 4** **and Fig. S2**). To conduct SEC-FRET analysis on the striatum, we pooled the striata of five mice into each single age group sample to reach sufficient quantities (3 mg) of total soluble protein for separation on the SEC column. Interestingly, full-length mhtt protein eluted in the 750 kDa fractions (peak 1; **Fig. 5A**) instead of the 1000 kDa fractions as observed in forebrain profiles suggesting a different composition of full-length mhtt protein complexes in the striatum. Western Blot analysis confirmed that full-length mhtt eluted at 750 kDa (**Fig. 5B**). Another observation is that the level of full-length mhtt protein did not significantly change with age (peak1, **Fig. 5A** **, Fig. S3A**). Fragments of mhtt eluted as two separate size pools at 250 kDa (peak 2a) and 160 kDa (peak 2b) and both pool sizes first slighlty increased during the first two months followed by their significant decrease starting at the age of 3 months (**Fig. 5A** **, Fig. S3A**). As compared to the age-dependent decrease of the 200 kDa pool in forebrain tissue (**Fig. 2B** **and** **Fig. 3A, B, C**), the age-dependent decrease of the two striatal mhtt fragment pools was much more profound (**Fig. 5A**). In accordance with the immunocytochemical data (**Fig. 4**), insoluble mhtt levels (MW8-Tb/MW8-D2 TR-FRET) showed very significant age-dependent increases in the striatum (**Fig. 5D**). Western blot analysis of the 250 kDa fractions (peak 2) revealed MW1-reactive mhtt fragments of around 85 kDa that were not detected with the 2B7 antibody (see comment for Fig.2D) (**Fig. 5B**). In addition, these mhtt fragments at around 85 kDa seem to undergo further processing with increased age and generate further fragments at 85 and 60 kDa (**Fig. 5C**). These results demonstrate that the nature of mhtt oligomeric fragments present in forebrain and striatum differs. Also, the inverse relationship between soluble oligomeric mhtt fragments and insoluble aggregates is far more dramatic in striatum as compared to the forebrain (**Fig. 5A, D** **and** **Fig. 2B** **,** **Fig. 3**).

Next, we used the antibody combination 2B7-Tb and MW8-D2 for the TR-FRET assay. This combination might be more specific for mhtt Exon1 fragments given the specificity of MW8 for a *de novo* generated C-terminal exon-1 epitope [8]. We did see a weak signal that overlapped with oligomers detected by the 2B7-Tb/MW1-D2 TR-FRET (**Fig. 5E** **and Fig. S3B**). Interestingly, the level of these 2B7-Tb/MW8-D2 soluble fragments increased slightly between 1 and 2 months of age before dramatically decreasing until 6 months of age when it reached a steady-state (**Fig. 5E** **and Fig. S3B**). Western blot analysis of soluble and insoluble brain protein fractions of 3, 8 and 12 month-old mice demonstrated that indeed, the mhtt fragments accumulate in insoluble aggregates (**Fig. 5F**). These findings support the idea that exon1 fragments rapidly form heteromeric oligomers containing different N-terminal fragments and that later-on, these are sequestered into IBs.

### Human Brain but not Fibroblasts Contain Soluble mhtt Fragments

**Figure 6:**
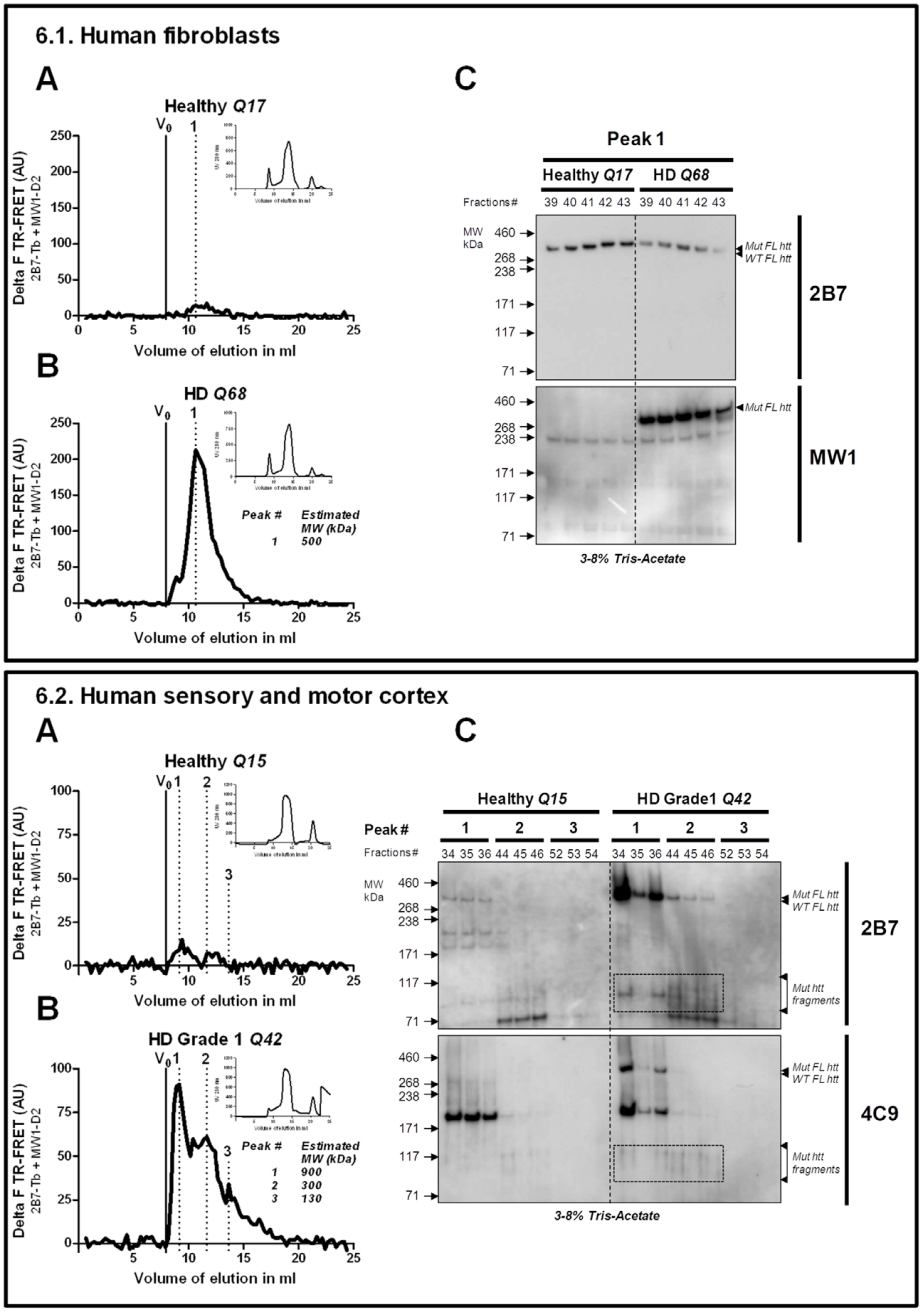
Detection of mhtt species in human samples using SEC-FRET technology. 6.1. SEC-FRET profiles with the UV absorbance in the upper right corner in human fibroblasts (A) Healthy (Q7), (B) HD (Q68) (C) Detection of mhtt full length protein (FL) in HD fibroblast by Western blot analysis using MW1 or 2B7antibodies 6.2. SEC-FRET profiles with the UV absorbance in the upper right corner in human *post mortem* sensory and motor cortex (A) Healthy, (B) HD grade1. (C) Detection of mhtt full length (peak1) and mhtt fragments (peak2) protein in HD human post mortem brain by Western blot analysis using 4C9 (human poly-proline) or 2B7antibodies.

To assess whether our findings may have clinical relevance for human HD, we first compared the SEC-FRET profiles of human fibroblasts from an HD donor with a polyQ stretch of 68Qs in the mhtt allele, with fibroblasts from a healthy donor carrying wildtype htt alleles. As expected, the HD but not the healthy donor-derived fibroblasts contained soluble mhtt species (**Fig. 6** **.1A, B**). The SEC-FRET profile of HD fibroblasts indicated the presence of full-length mhtt protein (peak at approximately 500 kDa) (**Fig. 6** **.1B**) but mhtt fragments were absent as confirmed also by Western blot (**Fig. 6** **.1.C**). These results in HD fibroblasts contrast with the SEC-FRET profile we obtained from human post mortem HD brain tissue of a grade 1 HD patient. This brain tissue revealed several soluble mhtt oligomeric species in 300 kDa size-fractions and that were not found in a healthy control (**Fig. 6** **.2A, B, C**). These findings demonstrate the presence of mhtt fragment-oligomers in the human HD brain that can be resolved using SEC-FRET.

## Discussion

In this study we combined SEC and TR-FRET to better resolve and define the formation, aggregation and age-dependent changes of endogenous mhtt protein complexes in HD cell and animal models. We found that endogenous full-length mhtt in ES-cell derived neurons, mice and human brain gives rise to mhtt N-terminal protein fragments that are present as soluble oligomers. We also found that the oligomers in the *Hdh*Q150 mouse brain can be detected at one month of age long before pathology is observed. In the *Hdh*Q150 mouse brain, the pool size of these soluble oligomers declines with age until about 8 months, from which time point onwards the pool-size seems to remain at steady- state. The decline of the soluble oligomer pool but not the levels of soluble full-length mhtt, inversely correlates with the formation and accumulation of insoluble mhtt aggregates. Finally, we demonstrated that the pool size of soluble mhtt oligomers is little or not influenced by mhtt gene dosage suggesting that the maximal size is reached and flux of mhtt molecules through this pool might be a key rate-limiting step in the formation of insoluble mhtt aggregates.

Our work provides, to our knowledge, for the first time an adequate and sensitive method under non-denaturing conditions for the detection and discrimination of populations of soluble mhtt oligomerized fragments and protein complexes generated by endogenous proteolytic cleavage of full-length mhtt in native settings in cells, mice and the HD patient brain. This is a new approach to our previous publications because its add a new biochemical dimension of oligomer vs aggregated and fragment vs full length relation of htt that may help to identify novel molecular pathways and molecules that are able to modify mhtt proteostasis and change disease course. The methodology we employed here may also facilitate the identification of brain region, species and cell-type specific mhtt protein interactions that may provide entry points for new HD therapies.

Relevant with respect to the use of clinical biopsy material and translational aspects related to measuring changes in mhtt protein in patients, we did not detect soluble mhtt oligomers or aggregates in human HD patient fibroblasts. This is in sharp contrast to their presence in all other samples we analyzed including mouse ES-derived neurons and *Hdh*Q150 mouse forebrain and striatum. This is also in sharp contrast with the analysis of human HD brain that also revealed striking signal of soluble mhtt oligomer formation compared to human brain control although future validation with more samples is here needed. One obvious explanation is that unlike the other specimens that mainly comprise non-mitotic cells, fibroblasts are mitotic cells. In proliferating cells, dilution of the soluble mhtt fragment-based oligomer pool may cause a drop in levels such that they no longer remain detectable using the TR-FRET assay. Note also that aggregation of mhtt in HD fibroblasts has not been observed and similar findings apply to many peripheral HD tissues with high contents of proliferating cells [8], [26]. Whether the lack of a soluble mhtt oligomer pool is mainly explained by the proliferative nature of fibroblasts or by cell-type specific processing will require additional experiments. Possibilities to address this question is to use mitotic-arrested fibroblasts, HD patient fibroblast-derived iPS cells, iPS-derived neurons and/or biopsies from human HD tissues including those with a high content of non-proliferating cells such as muscle. One goal of such studies would be to determine whether SEC-FRET analysis can identify oligomer pools in peripherial tissue that are suitable for biomarker studies and determine if these pools correlate with the soluble pool in the brain. Ultimately, this could lead to the identification of a relevant biomarker for HD.

The *Hdh*150Q mouse we used in this study closely geno- and phenocopies pathophysiological changes seen in the human HD brain [10]. Using immuno-precipitation, a recent study identified 14 different mhtt proteolytic N-terminal fragments in the brain of these mice [19]. The authors also provided evidence for MW8 detecting mainly a neo-epitope at the C-terminus of exon-1 and the formation of aggregates by such fragments [19]. Here, we confirmed that IBs in the *Hdh*Q150 mouse brain are detected by the MW8 TR-FRET assay and by MW8 immunostaining. Given the heterogeneous collection of N-terminal mhtt fragments generated in *Hdh*Q150 brains [19] it is perhaps surprising that the heterogeneity in size of soluble mhtt fragment-containing oligomers as detected by SEC-FRET analysis is low because most of the fragments elute in size-fractions of around 200 kDa. This seems to suggest that multiple mhtt fragments of different sizes form soluble heteromeric oligomers or insoluble aggregates.

The existence of exon-1 fragments has previously been described in *Hdh*150Q mice [19]. However, the levels of monomeric exon1 fragments in solution must be very low or absent at least in concentrations in the SEC fractions that no longer can be detected by the 2B7-MW1 TR-FRET. Note, even though this assay is about 10- fold more sensitive in detecting exon1 as compared to longer mhtt fragments or full length mhtt (**Fig1B**). Therefore, it seems likely that the aggregation-prone monomeric exon-1 fragments form very rapidly soluble oligomers or, alternatively, become sequestered into insoluble aggregates. The similar size of the soluble oligomer pool in homozygous and heterozygous mice is intriguing. One explanation for this observation could be that the flux rate of fragments sequestered into and out-of this pool is increased. Alternatively, newly generated mhtt fragments may escape this pool altogether because of its limited capacity and therefore instead flux straight into the insoluble aggregate pool.

One striking finding is that full-length mhtt-containing protein complexes in the striatum eluted at a different size as compared to those in forebrain suggesting that such soluble complexes have different brain region-specific and possibly also cell-type dependent compositions. Why such striking differences exist remains to be determined and awaits a further characterization of possibly variant mhtt N-terminal fragments and associated proteins in these complexes.

The classic model proposed for mhtt aggregation is based on a polyQ region-dependent nucleation and seeding leading to the formation of rigid, stable and fibrillar aggregates with atypical beta-sheet amyloid structure [27], [28], [29]. This model is proposed and mainly based on results obtained from *in-vitro* studies [27], [28], [29]. Analysis of HD brains has however provided little evidence for staining with specific amyloid dyes such as Congo Red or Thioflavin T [1] suggesting that mhtt aggregation in cells likely deviates from such a classical amyloid aggregation pathway model. Indeed, a recent study did point out that the aggregation pathway of mhtt fragments might be different *in-vivo* as compared to *in-vitro* [9], [30]. Using an elegant sedimentation velocity method to detect mhtt aggregates in cell lysates, these authors described an oligomeric mhtt pool that appeared to be at steady-state and rate-limiting to IB formation [10]. Consistent with these results, our SEC-FRET analysis revealed a broad peak fraction around 200 kDa that contains at least two different mhtt fragments in the same fractions and thus supports the hypothesis that heteromeric complexes form *in-vivo*. Already mentioned before, we can not exclude the possibility that soluble mhtt fragment oligomers are not intermediates in IB formation but rather represent off-pathway species that are directly sequestered in IBs [10], [28], [31]. However, in our longitudinal SEC-FRET profiling study of heterozygous mice we showed that an inverse correlation exists between the pool-size of soluble mhtt fragment- oligomers and the increase in insoluble aggregates. This seems to suggest that aggregate formation is directly linked to the pool of soluble mhtt fragment-oligomers.

The question arises whether the mhtt species we detected using the 2B7-Tb MW1-D2 TR-FRET assay indeed represent mhtt fragment-oligomers? This TR-FRET assay detects only mutant and not wildtype htt molecules and only mhtt with conformations that expose simultaneously the 2B7 and the MW1 epitope (i.e. the first 17aa residues and the polyQ stretch) [8]. Other mhtt conformations in which one of these epitopes is buried are not detected by this TR-FRET assay [8]. It is likely that both epitopes are exposed in mhtt monomers that may adopt different misfolded conformations [8], [32], [33], [34]. Nonetheless, the SEC-FRET profiles provided little or no evidence for the presence of soluble monomers. In contrast, the identification, detection and co-elution of multiple N-terminal mhtt fragments in single 200 kDa fractions clearly indicates that the 2B7-MW1 TR-FRET does detect mhtt fragments that are part of larger oligomeric complexes in which these mhtt molecules expose both the polyQ and the N-terminal 17 amino acid 2B7 epitope. Support for oligomers that expose polyQ-epitopes comes from a study where gold-labeled MW1-antibodies captured mhtt oligomers from *Hdh*Q150 cortices shown by immuno-electron microscopy [35]. These oligomers likely do not have amyloid character because the polyQ epitope is not buried and inaccessible to the Abs as it is in classical beta-sheet conformations. Therefore, it seems that soluble mhtt complexes contain also other factors, e.g. chaperones, that might prevent beta-sheet formation and stabilize different mhtt conformational states [10], [34], [36], [37].

Previous studies including our recent publication have provided evidence of inverse correlations between the amount of aggregated and soluble mhtt [8], [35], [38] but they did not provide clues as to which soluble mhtt protein species is prone and a possible precursor to aggregates. Our study provides strong hints that specifically a pool of soluble mhtt fragments and not full-length mhtt protein are causally linked to aggregate formation thereby further strengthening the hypothesis that N-terminal fragments are directly responsible for the formation of IBs rather than full-length mhtt protein undergoing post-aggregation cleavage [3]–[7].

**Figure 7:**
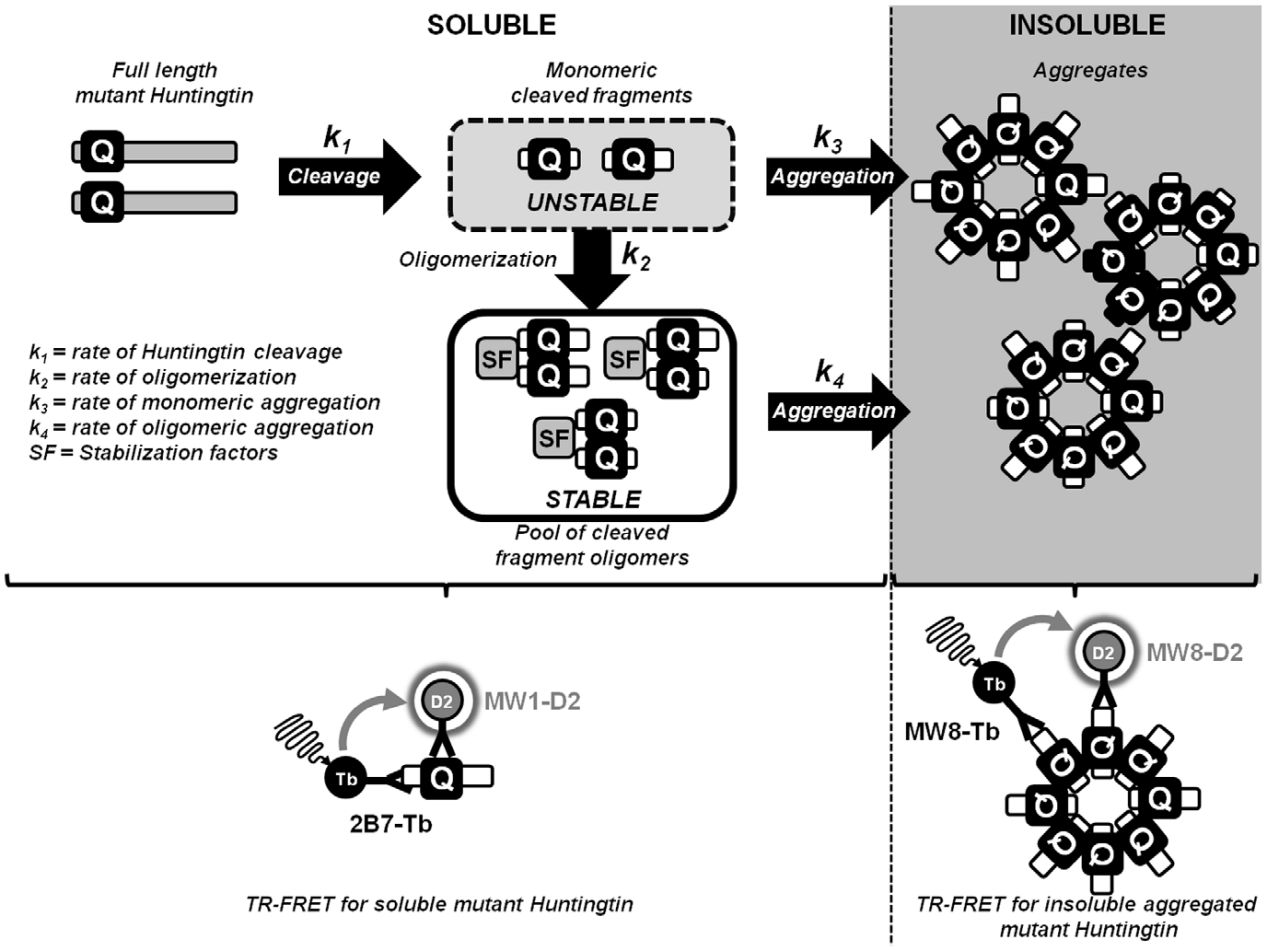
A model of how mutant htt aggregates in cells. After proteolytic cleavage of the full-length mutant htt protein, free monomeric N-terminal fragments are extremely short-lived, likely because the expanded polyQ region confers great conformational flexibility but misfolded states at the same time (k1). These facilitate unspecific protein-protein interactions that compromise cell function and viability. As a first line of defense, cells may therefore capture mutant htt monomeric fragments rapidly into soluble oligomers (k2). The pool size capacity of soluble oligomers may be limited by the availability of stabilization-factors (SF) of the protein quality control (chaperone, autophagy, proteosome, etc.). Chronic saturation of the soluble oligomer pool would likely increase the flux of mutant htt fragments into and out of the soluble oligomer pool into insoluble aggregates, perhaps the last resort and coping strategy cells can count on to prevent mutant htt fragments from impairing cell function (k3 and k4).

Our SEC-FRET analysis of homozygous *vs* heterozygous mice suggested that the pool size capacity of soluble oligomers may be limited. Our results also indicate that a chronic saturation of the soluble oligomer pool happens at a much younger age in homozygous as opposed to heterozygous *Hdh*Q150 mice. Therefore, the increased mhtt gene dosage in homozygous *Hdh*Q150 mice likely increases the flux of mhtt fragments into and out of the soluble oligomer pool and into insoluble aggregates. Perhaps this mechanism is a coping strategy that cells have to prevent mhtt fragments from impairing cellular function. In this study we did not address the issue of oligomer toxicity per se. The fact that the oligomer pool is detected already in brains of one month-old *Hdh*Q150 mice, long before any histopathophysiological phenotypes appear, certainly seems in line with the hypothesis that this fragment-oligomer pool may have a protective role preventing misfolded oligomers from exerting cellular toxicity. We therefore propose a working hypothesis (**Fig. 7**) that is based on a limited capacity of the pool size of soluble oligomers as suggested by our SEC-FRET analysis of homozygous versus heterozygous mice. The size of the soluble oligomer pool might depend on the availability of stabilization-factors (SF) of the protein quality control machinery including chaperones and components of the autophagy and proteosome pathways. Chronic saturation of the soluble oligomer pool, something that likely happens at a much younger age in homozygous as opposed to heterozygous *Hdh*Q150 mice, would likely increase the flux of mhtt fragments into and out of the soluble oligomer pool into insoluble aggregates. The latter are perhaps the last resort cells have as coping strategies to prevent mhtt fragments from impairing cell function (**Fig. 7**). During aging, the capacity to form protective soluble oligomeric complexes seems to decline as our analysis in *Hdh*Q150 heterozygote mice have shown. This decline might be the result of age-dependent reductions observed for example in chaperone expression [39] or the activity of degradation systems [40], [41]. If the generation of mhtt fragments does not decline, the soluble oligomer pool will gradually have less buffering capacity to capture newly generated mhtt fragments. At the same time, this likely increases the risk of undesirable protein-protein interactions that compromise cell function and viability (**Fig. 7**). From a therapeutic point of view it will be highly interesting to see if modulators such as macroautophagy-inducers or pharmacological chaperones that might increase or decrease the size of the soluble oligomer pool will indeed slow or exacerbate disease progression.

In conclusion, we presented a SEC-FRET based analysis that provided novel insights into the formation of soluble oligomers of mhtt fragments under native conditions. Most striking, mhtt fragments form a soluble oligomer pool which precedes and inversely correlates with the deposition of insoluble aggregates. We believe that the SEC-FRET technology will be useful to address multiple unanswered questions regarding mhtt proteostasis and perhaps it opens new routes to finding modulators of disease in HD and other neurodegenerative protein misfolding diseases [42], [43].

## Supporting Information

Figure S1 **(A)** SEC elution profiles of marker proteins lead to linear correlation. The superdex 200 10/30 column was calibrated with Gel Filtration Marker Kit for Protein Molecular Weights 29,000–700,000 Da (Sigma # MWGF1000). Proteins were diluted in PBS and injected into the column. The volume of elution for each protein marker Ve was normalized to the elution volume obtained with Blue dextran (Dead volume  =  V0). Ratio Ve/V0 were plotted with their respective molecular weight logarithm to obtain a linear regression. **(B)** Total supernatant western blot analysis to detect mutant htt fragments with 2B7 antibody before injection onto SEC column. Western Blot analysis of wild type, Hdh Q150/− and Q150/Q150 mice forebrain supernatant extracts after ultracentrifugation at 100.000 g for 30 min (30 µg/well). 2 bands are detected: the upper band is specific for mutant htt fragment at around 80 kDa; the lower band is unspecific**.**
(PDF)

Figure S2 **Quantitative analysis of aggregate size.** Sizes of aggregates were calculated with the CellF software (Soft Imaging Systems/Olympus). About 100 IBs in 3 images/timepoint were analyzed. Significance was determined by Mann Whitney test, p value <0.0001 (one-tailed).
(PDF)

Figure S3 (A) Quantification of the area under the curve (AUC) for the peaks 1, 2a and 2b for the different ages analyzed.(B) SEC-FRET profile of HdhQ150 mice brains with TR-FRET antibody combination 2B7-Tb/MW8-D2 at 3, 4, 6 and 8 months.
(PDF)
