# Supplementary figures and images for: Fragments of HdhQ150 Mutant Huntingtin Form a Soluble Oligomer Pool That Declines with Aggregate Deposition upon Aging
Source: PLoS One. 2012 Sep 12;7(9):e44457. doi: 10.1371/journal.pone.0044457 (PMC3440421; doi:10.1371/journal.pone.0044457)

**A**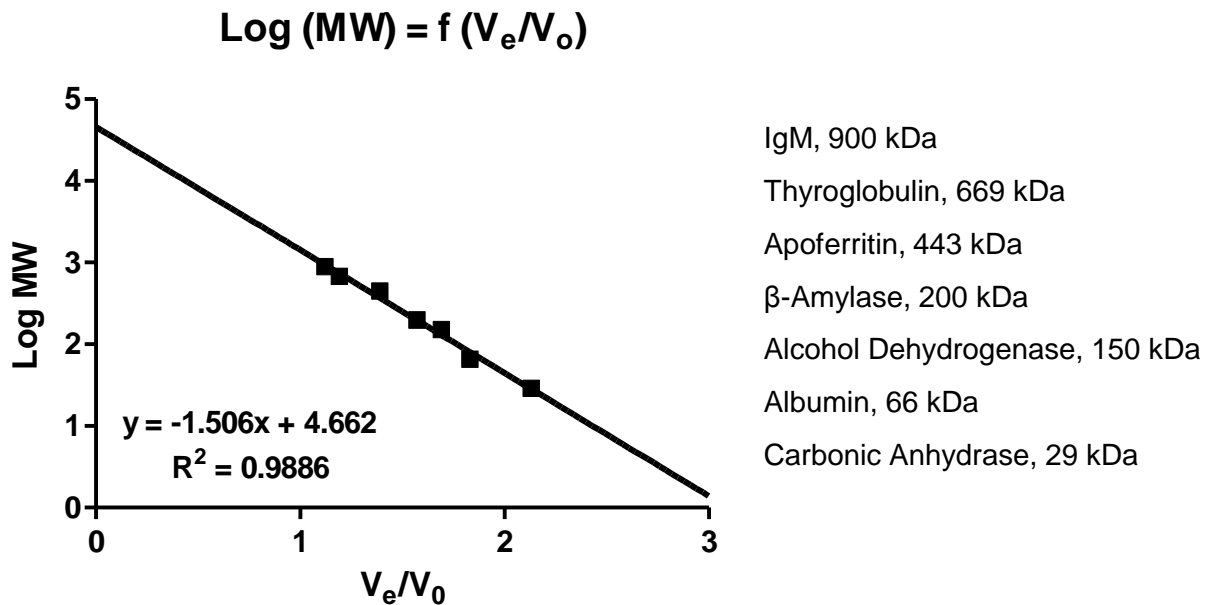**B**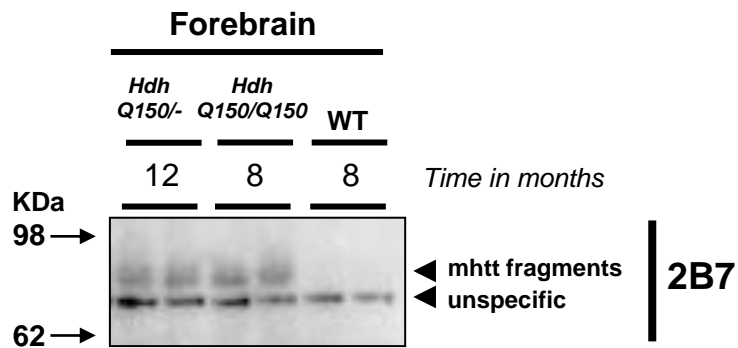**Figure S1**

Supplement: Figure S1 — (A) SEC elution profiles of marker proteins lead to linear correlation. The superdex 200 10/30 column was calibrated with Gel Filtration Marker Kit for Protein Molecular Weights 29,000–700,000 Da (Sigma # MWGF1000). Proteins were diluted in PBS and injected into the column. The volume of elution for each protein marker Ve was normalized to the elution volume obtained with Blue dextran (Dead volume = V0). Ratio Ve/V0 were plotted with their respective molecular weight logarithm to obtain a linear regression. (B) Total supernatant western blot analysis to detect mutant htt fragments with 2B7 antibody before injection onto SEC column. Western Blot analysis of wild type, Hdh Q150/− and Q150/Q150 mice forebrain supernatant extracts after ultracentrifugation at 100.000 g for 30 min (30 µg/well). 2 bands are detected: the upper band is specific for mutant htt fragment at around 80 kDa; the lower band is unspecific. (PDF) [file pone.0044457.s001.pdf]

aggregate size in the striatum of  
 $Hdh^{Q150/+}$  mice

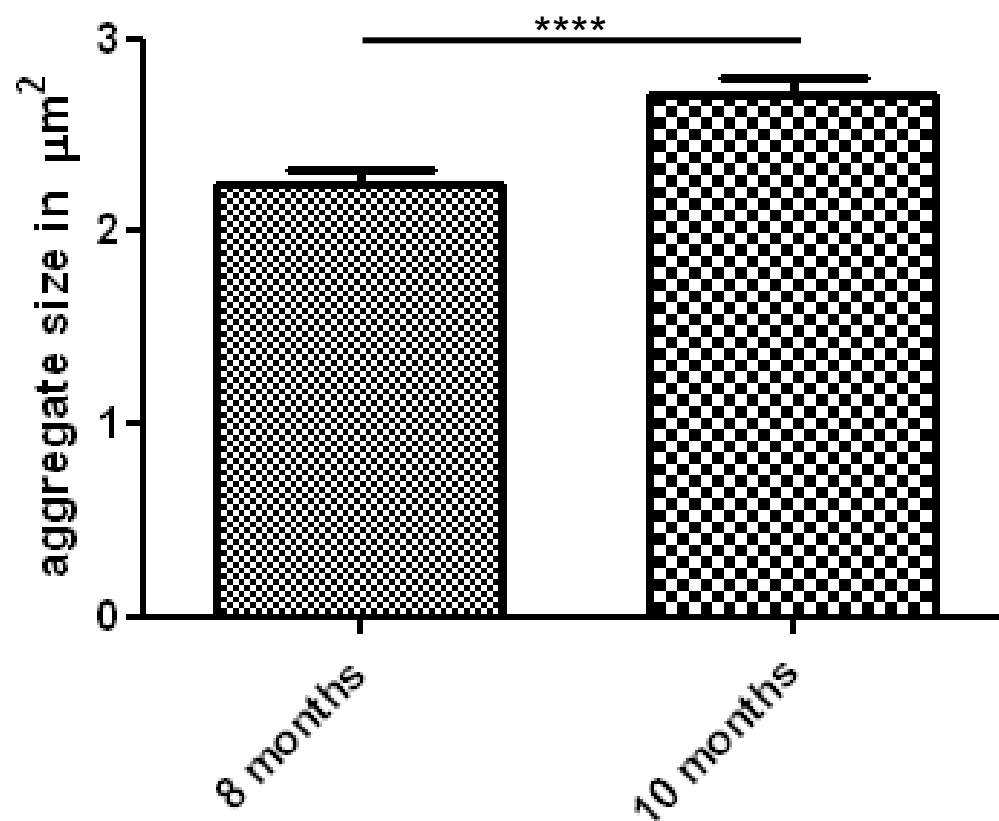

Figure S2.

Supplement: Figure S2 — Quantitative analysis of aggregate size. Sizes of aggregates were calculated with the CellF software (Soft Imaging Systems/Olympus). About 100 IBs in 3 images/timepoint were analyzed. Significance was determined by Mann Whitney test, p value <0.0001 (one-tailed). (PDF) [file pone.0044457.s002.pdf]

**A**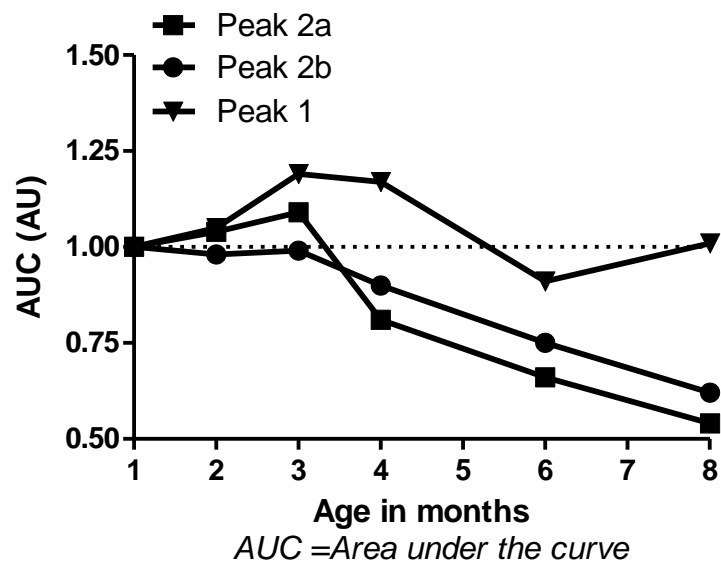**B**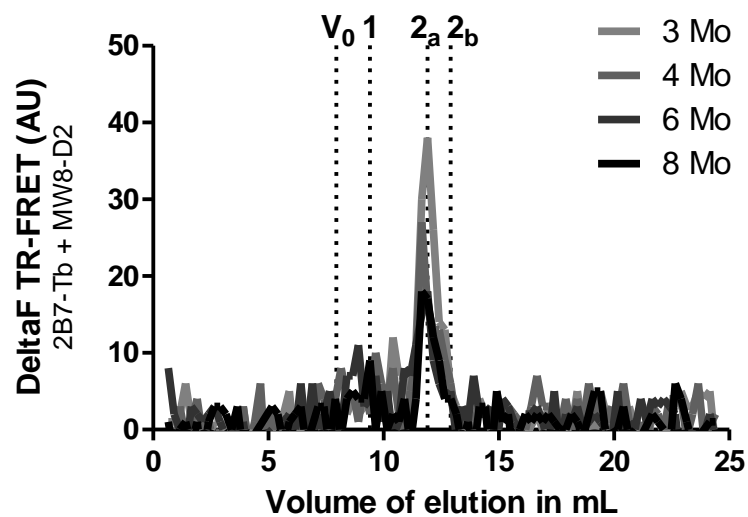**Figure S3**

Supplement: Figure S3 — (A) Quantification of the area under the curve (AUC) for the peaks 1, 2a and 2b for the different ages analyzed.(B) SEC-FRET profile of HdhQ150 mice brains with TR-FRET antibody combination 2B7-Tb/MW8-D2 at 3, 4, 6 and 8 months. (PDF) [file pone.0044457.s003.pdf]
